# Epigenetic age predictors for non-invasive assessment of human skin

**DOI:** 10.1038/s41514-025-00314-0

**Published:** 2025-12-12

**Authors:** Angel Menendez Vazquez, Dimitris Katsanos, Miruna Vasile, Andrew Graham, Victoria Dyster, Shakiba Kaveh, Mahdi Moqri, Cristiana Banila

**Affiliations:** 1 Mitra Bio, Translation and Innovation Hub, London, UK; 2 Division of Genetics, Department of Medicine, Brigham and Women’s Hospital, Harvard Medical School, Boston, MA USA

**Keywords:** Biological techniques, Biomarkers, Genetics

## Abstract

Skin is both the most visible and most environmentally exposed organ, with apparent aging phenotypes. DNA methylation clocks faithfully capture the progression of aging, but so far have been limited to training on abundant in vitro material or invasively collected samples to generate narrow methylomes using microarray platforms. Here, we demonstrate that skin biological age can be measured directly from a person’s face with superior accuracy, using non-invasive tape-stripping. We developed two clocks, MitraSolo, based on single CpGs, and MitraCluster, on regions, trained on the largest enzymatic methyl-sequencing dataset of human epidermis (*n* = 462). Our models were validated on independent, longitudinal, and external datasets and were compared against established clocks. They predict age accurately, with an error of approximately 4 years, outperforming others on epidermal samples. They maintain high accuracy at low sequencing depths, enabling cost-effective scalability and show intra-individual prediction variation <2 years, highlighting their reproducibility. Their predictive capacity generalised across anatomical sites, conversion and sampling methodologies and on in vitro material. They also successfully captured the rejuvenating effects of Yamanaka factor treatment. MitraSolo and MitraCluster represent a new class of epigenetic clocks optimised for human skin with characteristics that support their use in clinical research, intervention monitoring, and skincare innovation.

## Introduction

As the most visible organ affected by aging, the skin is uniquely positioned at the intersection of intrinsic biological decline and extrinsic environmental stress. Over time, exposure to ultraviolet (UV) radiation, pollution, inflammation and physical wear can impair the structure and function of the skin. Due to its rapid cellular turnover and constant contact with the environment, the skin not only reflects visual changes associated with aging but may also play a role in broader age-related processes in the body^1^. A robust and tissue-specific biomarker of biological skin age is urgently needed, not only for understanding the aging process but also for evaluating the effects of rejuvenation therapies and topical interventions in clinical and consumer settings.

DNA methylation is one of the most promising biomarkers of aging. It changes in a predictable manner across time, is sensitive to environmental and therapeutic influences, and may even mediate age-related phenotypic drift^2^. Epigenetic age predictors or clocks, built from these methylation changes, have demonstrated utility in quantifying biological age and tracking the efficacy of diverse interventions. For example, multi-modal lifestyle programmes, including nutritional changes, stress reduction, and exercise, have been shown to reduce epigenetic age^3^. Exercise alone has been associated with age deceleration in multiple tissues^4^, and pharmacological interventions such as rapamycin have shown similar trends^5^. As a result, recent consensus frameworks have identified DNA methylation clocks as central tools for guiding and assessing aging interventions^3,6^.

Despite their promise, existing clocks are subject to key limitations. The most widely used ones include the Horvath Pan-Tissue, Skin & Blood, Hannum and PhenoAge models, and are developed from blood or cell culture material, using microarray platforms such as Illumina’s 450K or EPIC arrays^7,8^. These platforms require an abundance of material for DNA methylation measurement and, therefore, struggle with the limited nature of non-invasive in vivo samples. As a result, they often do not accurately represent the cellular makeup and environment of in vivo tissues. In addition, microarray platforms capture fewer than 3% of genomic CpGs, are semi-quantitative in nature, and predominantly target CpG islands and promoter regions^9^. They treat each CpG as an independent variable, ignoring co-methylation patterns across genomic regions that may better reflect coordinated biological aging. Together, these limitations constrain the translational utility of existing array-based clocks, underscoring the need for sequencing-based, region-aware models^10,11^.

Recent advances in sequencing-based epigenetic clocks have begun to overcome some of these technical challenges. Region-based clocks such as those targeting polycomb repressive complex 2 (PRC2) sites have demonstrated improved accuracy and biological relevance^12^, and models like BS-Clock, trained on high-resolution bisulfite sequencing, have shown enhanced precision across several tissue types^13^. However, these clocks are not tailored to skin and are typically trained on internal tissues or biopsy-derived samples that contain heterogeneous mixtures of cell types.

Tape-stripping offers a scalable, non-invasive alternative for collecting epidermal DNA from human skin. Previously, we showed that tape-stripped samples are suitable for high-resolution methylome profiling and retain tissue-specific signatures, enabling molecular studies of cutaneous aging in vivo^14^. Yet, existing epigenetic clocks were not designed to work with tape-stripped skin, largely because most models are trained on easily accessible tissues like blood or dermal biopsies, not on in vivo, keratinocyte-rich epidermal samples with limited material. To address this gap, we propose a two-part solution: first, establishing the largest enzymatic methyl-sequencing (EM-seq) database using high-quality DNA from non-invasively collected human skin, and second, developing skin-specific predictive models. As such, we present MitraSolo and MitraCluster, the first epigenetic age predictors developed from the same epidermal EM-seq database. MitraSolo leverages individual CpG methylation features, while MitraCluster uses region-level methylation, aggregating co-correlated CpG sites into functionally coherent signals.

This study presents three key findings. First, leveraging the largest EM-seq skin methylome database to date, we introduce the first epigenetic clocks trained on non-invasively collected human skin samples, which outcompete existing array- and sequencing-derived models when applied to skin samples. Second, we show that the region-based modelling strategy enables robust predictions even at ultra-low sequencing depths, proving that methylation sequencing can be both highly accurate and cost-efficient for biological age estimation. Third, despite being trained in enzymatically converted tape-stripping skin samples, the clocks generalise across sampling methods, tissues, platforms, and experimental contexts, retaining accuracy in diverse sample types and capturing age reversal signatures in rejuvenation settings.

## Results

### The skin-specific epigenetic age predictors accurately predict chronological age in human skin samples

**Fig. 1:**
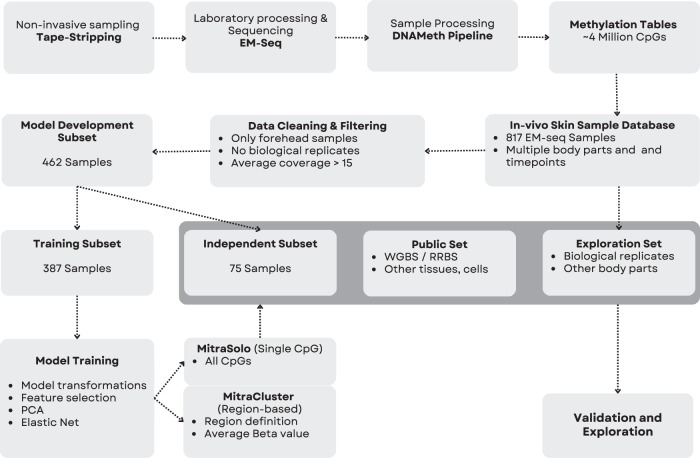
Overview of the MitraClocks epigenetic age predictors development pipeline. An illustration and outline of the structure of the Mitra Bio database, the sample filtering process, and the creation of two predictive models: a single CpG-based model (MitraSolo) and a region-based model leveraging clusters of age-correlated CpGs (MitraCluster).

**Fig. 2:**
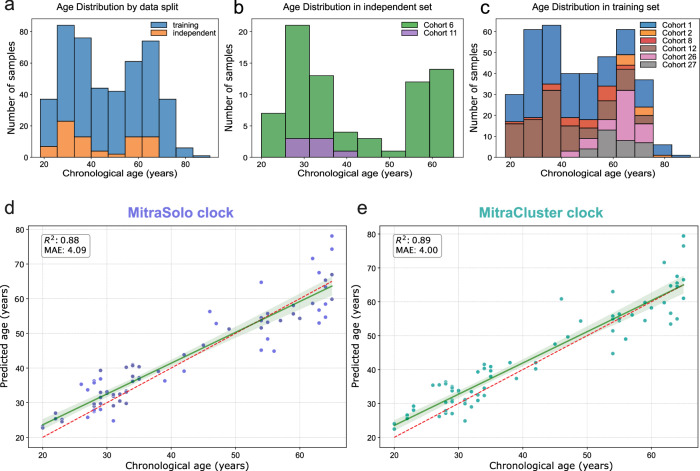
The age predictor models’ sample distribution and prediction accuracy. **a** Histogram showing age distribution of all samples used in Mitra clocks development, split into training (blue) and independent (orange) sets. **b** Age distribution of the independent dataset stratified by data cohorts included. **c** Age distribution of the training dataset stratified by data cohorts included. Cohorts in (**b**, **c**) represent separate sampling and sequencing batches. **d**, **e** Predicted versus chronological age in the independent test set for the single-CpG model, MitraSolo (*R*^2^ = 0.88, MAE = 4.09) (**d**) and the region-based model, MitraCluster (*R*^2^ = 0.89, MAE = 4.00) (**e**), demonstrating high accuracy in both models. The independent set samples cover, on average, 98.1% of the MitraSolo CpGs and 97.9% of the MitraCluster CpGs.

The skin, as the body’s primary interface with the external environment, exhibits visible signs of aging. To capture the molecular signatures of this process in vivo, we developed skin-specific DNA methylation clocks using non-invasively collected epidermal samples. We profiled the methylomes of 590 individuals via tape-stripping, generating a high-resolution sequencing dataset spanning approximately 4 million CpG sites per sample (Fig. 1). Our dataset was divided into a training set and an independent validation set with comparable distributions of age and sex across both groups (Figs. 1 and 2a–c).

Utilising a PCA-Based ElasticNet Machine Learning model, we trained the clock using a total set of 3,830,117 CpGs as input. The resulting model, named MitraSolo, is, to our knowledge, the first DNA methylation age estimator relying on sequencing of enzymatically converted DNA. After feature reduction, it uses methylation values from 3831 CpGs. We validated the model on an independent cohort by comparing predicted versus chronological age, demonstrating its accuracy and generalisability beyond the training data. The MitraSolo clock showed high prediction accuracy across a wide range of skin ages, with a mean absolute error (MAE) of 4.09, median absolute deviation (MAD) of 3.14, root mean squared error (RMSE) of 5.25 and an coefficient of determination (*R*^2^) score of 0.88 (Fig. 2d), which is at least on par with the best in class methylation clocks for skin^15^.

To improve prediction accuracy, we exploited the high-resolution nature of sequencing data to train a region-based model. Since neighbouring CpG sites often exhibit correlated methylation patterns and form co-methylated regions, modelling them jointly has been shown to capture more biologically relevant variation and reduce noise^16,17^. We took advantage of this by defining regions across the genome based on levels of co-correlation of neighbouring CpGs to age. We then calculated average methylation values from those CpGs for every region, using them as features to train a region-based clock—MitraCluster. After feature reduction, this model uses 931 regions of at least 3 CpGs each, resulting in 14,089 CpGs being used for the calculation. Using the same independent test set as MitraSolo, we observed a reduced MAE of 4.00, MAD of 2.96, RMSE of 5.23 and an *R*^2^ score of 0.89 (Fig. 2e). Overall, both clocks show robust prediction metrics on an independent dataset, with the region-based clock slightly outperforming the individual CpGs model. We also looked at whether our models showed any variation in prediction accuracy driven by sex, using our validation dataset. We found very similar metrics for both sexes and both clocks (Supplementary Fig. 1a, b), showing that at least on our validation dataset, sex does not appear to impact prediction accuracy. Tracking of the participants’ Fitzpatrick score also did not point to obvious performance biases, but a broader dataset will be required to adequately address this possibility (Supplementary Fig. 1a, b and Supplementary Table 1).

Finally, we decided to look at the localisation of the CpGs selected by our clocks, both relative to various genomic features, but also in the context of CpG density. The significance of these overlaps was assessed with a Monte Carlo-based approach, simulating matched randomised distributions within the capture panel-defined workable genome. We found that for both of our clocks, our CpGs were statistically significantly enriched in gene exons and within CpG islands (Supplementary Fig. 1c, d). They were also both significantly depleted from gene introns, 3’ UTRs, CpG shores, shelves and intergenic regions (Supplementary Fig. 1c, d). Interestingly, both clock CpGs are slightly depleted from promoters, which tend to be associated with CpG islands. Together with the above findings, this points to a preference for selecting CpGs within coding sequences covered by CpG islands. As an additional exploration of the clock CpGs, we extracted common sites across both of our models, assigned them to the closest gene and ran a pathway enrichment analysis on them. A list of significantly enriched pathways is presented in Supplementary Table 2. A variety of ontology terms related to gene regulation and development have been found to be enriched, however, the true functional value of these CpGs is difficult to determine at this stage, as previously described^18^.

### The skin-specific epigenetic age predictors demonstrate robustness to low sequencing depth and high reproducibility across biological replicates

**Fig. 3:**
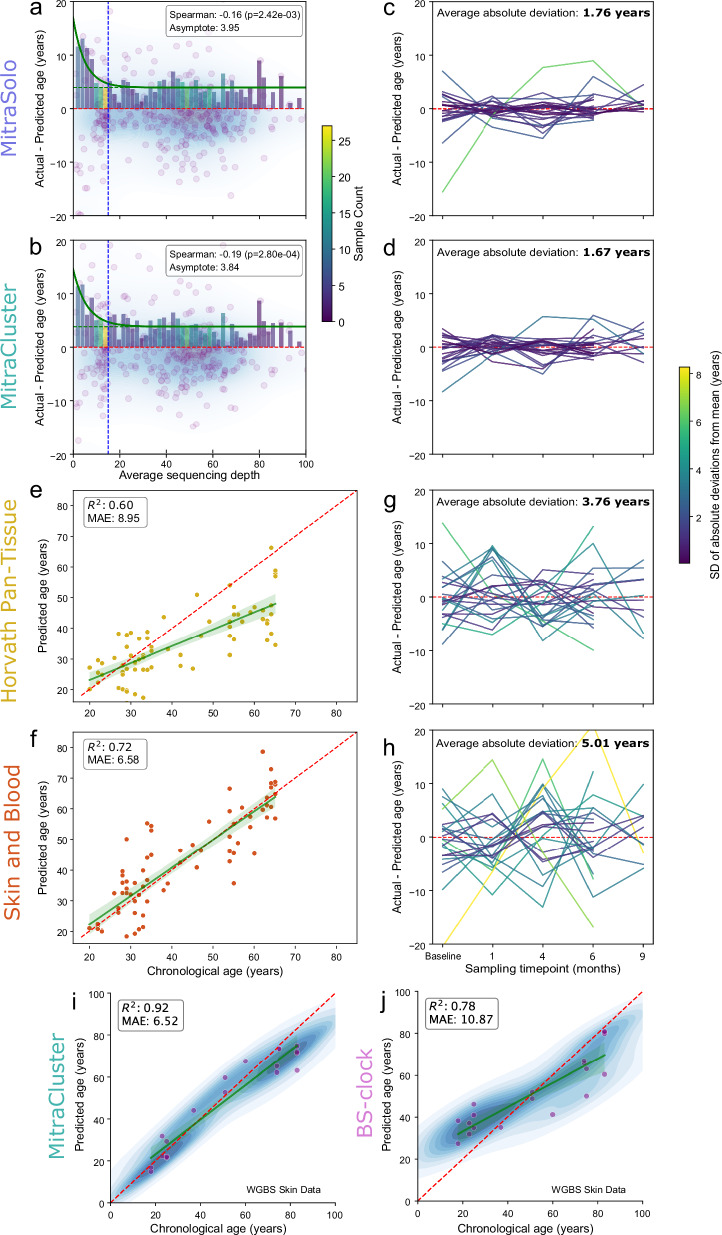
The skin-specific age predictors demonstrate sequencing depth robustness and superior prediction accuracy, and reproducibility compared to array-based clocks. Relationship between sequencing depth (average CpG coverage) and Δage for MitraSolo (**a**) and MitraCluster (**b**). Both models maintained stable accuracy down to 10× coverage, with Δage values reducing as coverage increased to an asymptote of 3.84 and 3.95 years, respectively, with low inverse correlations between coverage and Δage (Spearman *ρ* = −0.19 and −0.16, respectively). The asymptote curve captures absolute Δage. For individuals with multiple samples spanning 9 months, the difference in age prediction between each time point versus the average prediction of the individual was calculated and plotted over the nine months for MitraSolo (**c**) and MitraCluster (**d**). This is used as a measure of consistency across the predictions. Both models show minimal intra-individual variation over time, with an average absolute deviation from the individual mean of 1.76 and 1.67 years, respectively, coupled with a low standard error of the Mean of 1.04 and 1.25. Age prediction performance of Horvath’s Pan-Tissue (**e** MAE = 8.95, *R*^2^ = 0.60) and Skin & Blood clocks (**f** MAE = 6.58, *R*^2^ = 0.72) on the independent dataset. Longitudinal age prediction consistency (as in (**c**, **d**)) for the same individuals using the Horvath Pan-Tissue (**g**) and Skin & Blood (**h**) clocks reveal greater prediction noise and less temporal stability, with average absolute individual deviation from the mean of 3.73 and 5.02, and standard error of the Mean of 2.51 and 3.22 years, respectively. Age prediction accuracy on WGBS skin samples by MitraCluster (**i**) outperformed BS-Clock (**j**) with a MAE of 6.52 vs. 10.87. The WGBS skin data covers, on average, 86.2% of the MitraCluster clock CpGs.

Next, we assessed whether the prediction accuracy of our models was affected by sequencing depth. Unlike microarray data typically used for clock training, methylation estimates from sequencing are thought to be less reliable at low coverage, particularly below ~100× per CpG^9,19^. Our entire facial skin database spans a wide range of sequencing depths covering our clock sites with an average of around 61.8× (S.D: 49.2) for MitraSolo and 57.7× (S.D: 45.7) for MitraCluster (Supplementary Fig. 1a, b). To explore this, we utilised 269 facial skin samples that were not used in model training or testing due to low coverage or to avoid biological replicates, with average CpG coverage ranging from 1.02 to 396×. This dataset was used to explore the relationship between coverage and the Δage rate of the models. Both clocks demonstrated strong robustness to sequencing depth, with high errors at extremely low levels of coverage, but showed exponential improvement with an asymptote for the mean absolute Δage of 3.95 for MitraSolo and 3.84 for MitraCluster (Fig. 3a, b). The MAE for samples whose CpG sequencing depth ranges from 15× to over 150× is 3.99 for MitraCluster and 4.10 for MitraSolo. Notably, even at sequencing depths as low as 10×, which is below the threshold used during training, the models maintained a mean Δage of under 5 years (Fig. 3a, b).

To assess if the prediction accuracy begins to show sequencing depth robustness >9× due to the training set thresholding, we trained 2 additional versions of the models with depth filtering at 5× and 30×. In all cases, the age prediction accuracy approaches optimal levels for sequencing depths around 10× and above (Supplementary Fig. 2a–d). These findings support that our models can perform successful predictions at substantially shallower sequencing depths (i.e. 10×) than previously considered essential for DNA methylation sequencing data^9,19^. Moreover, when comparing the performance of our two clocks, we find that the region-based model shows consistently smaller Δage values, particularly at low sequencing depths, with an average absolute Δage of 4.94 for samples between 5× and 14× coverage for MitraCluster in comparison to 5.01 for MitraSolo (Fig. 3a, b). This supports the hypothesis that our region-based clock is more robust, as noise from sequencing depth differences is minimised across regions of CpGs.

Building on the finding that our models’ accuracy remains stable across a range of sequencing depths, we next assessed prediction reproducibility. Leveraging longitudinal replicate samples from our broader database, collected from the same individuals over a 9-month period (*n* = 76). We generated predictions at each time point and normalised them to each individual’s mean to track intra-individual consistency over time. (Fig. 3c, d). MitraSolo showed an average deviation of 1.76 years with a standard error of 1.25, while MitraCluster demonstrated even greater reproducibility, with an average deviation of 1.67 years and a standard error of 1.04. These low fluctuations, well below the overall model error, indicate high biological repeatability. The robustness of MitraCluster in longitudinal sampling makes it valuable for tracking skincare interventions. For these reasons, in the rest of this study, we focus on MitraCluster.

### MitraCluster outperforms existing epigenetic age predictors on sequencing-derived DNA methylation data from the skin

We compared the performance of the Mitra clocks to established epigenetic age estimators, including Horvath’s Pan-Tissue and Skin & Blood clocks, PhenoAge, Hannum, and BS-Clock^13,15,20–22^. Except for BS-Clock, all were trained on array data from ex vivo/cell samples, while our models rely on sequencing from non-invasively collected skin. To enable comparison, we transformed our data to microarray format and ran predictions on the same independent validation set. MitraSolo and MitraCluster outperformed all four microarray-derived clocks in accuracy (Fig. 3e, f and Supplementary Fig. 3a, b). Among microarray clocks, Skin & Blood performed best (MAE = 6.58; *r* = 0.72) (Fig. 3f), yet both that and the Pan-tissue clock showed weaker longitudinal reproducibility than our models, with prediction deviations of 5.01 and 3.76 years, respectively, versus MitraCluster’s 1.67 (Fig. 3g, h).

Directly evaluating MitraSolo and MitraCluster on microarray datasets is not feasible due to minimal CpG site overlap. Our models were trained on over 3.8 million CpGs, compared to only 937,690 on EPIC v2, 486,427 on HM450, and 27,578 on 27K arrays. Consequently, just a small fraction of our clock features is represented on these platforms (e.g. for MitraSolo: 6.92% on EPIC v2, 5.32% on HM450, 0.23% on 27K; for MitraCluster: 8.81%, 6.96%, and 0.39%, respectively), precluding meaningful prediction on array data. To address this limitation, we retrained skin-specific PCA-based versions of Horvath’s Pan-Tissue and Skin & Blood clocks using their original CpG features but fitted to our tape-stripped epidermal training dataset. When applied to our independent validation set, these retrained clocks showed improved performance (MAE = 7.58 for Pan-Tissue and 4.69 for Skin & Blood; Supplementary Fig. 3d–e) compared to their original versions. However, both were still outperformed by MitraSolo (MAE = 4.09) and MitraCluster (MAE = 4.00), highlighting the advantage of building clocks natively from high-resolution, skin-specific sequencing data. We further evaluated these retrained clocks, alongside their original versions, on an external epidermal microarray dataset to confirm their relative performance in an independent, array-based setting^23^. Interestingly, the Skin & Blood clock versions trained on our sequencing-based, tape-stripping samples outperformed the original model on these microarray-derived samples (Supplementary Fig. 3f–i). This shows that our dataset captures the effects of aging on human skin with greater accuracy, leading to the improved performance of this skin-specific clock even when making predictions on microarray data.

Beyond the comparison to the well-established microarray clocks, we looked to compare our models to a more similar tool, like BS-clock^13^, since our database is constituted of sequencing-based data. We conducted a two-part comparison with the publicly available BS-Clock model and its training dataset^13^. Only the version of BS-clock trained on blood is readily available for use on external datasets and is reported to show modest performance in skin biopsies (*R*^2^ ≈ 0.40)^13^. When applied to our epidermal EM-seq validation dataset, BS-Clock demonstrated minimal predictive capacity (MAE = 14.56 years, *R*^2^ = 0.03; Supplementary Fig. 3c), likely due to its blood-derived training set and limited site overlap with the methylome target panel we utilise (47.03% CpG coverage). Its poor accuracy on enzymatically converted, non-invasive epidermal samples suggests limited tissue and methodological generalisability. As such, we consider array-based clocks like Horvath’s Pan-Tissue and Skin & Blood more relevant comparators for our platform.

For the next step, we applied MitraCluster to BS-Clock’s own publicly available WGBS skin biopsy dataset (*n* = 19). For this comparison, we utilised the prediction values reported using the skin-trained version of BS-Clock. Despite differences in sample type, conversion chemistry, and platform, MitraCluster achieved markedly better performance (MAE = 6.52 years) than BS-Clock on its own data (MAE = 10.87 years) (Fig. 3i, j). MitraCluster achieves this accuracy in skin predictions even though the ex vivo BS-clock skin samples have been gathered with a variety of sampling methodologies and from various body parts other than the face. MitraCluster also outperformed the original and newly retrained Skin & Blood and Pan-Tissue clocks on this external dataset (Supplementary Fig. 3j–m), further supporting that its superior performance is not dataset- or platform-dependent. These findings underscore MitraCluster’s cross-platform robustness and its enhanced sensitivity to true biological aging signals in skin.

### The regional model is generalisable across sampling locations, tissues and cell types

**Fig. 4:**
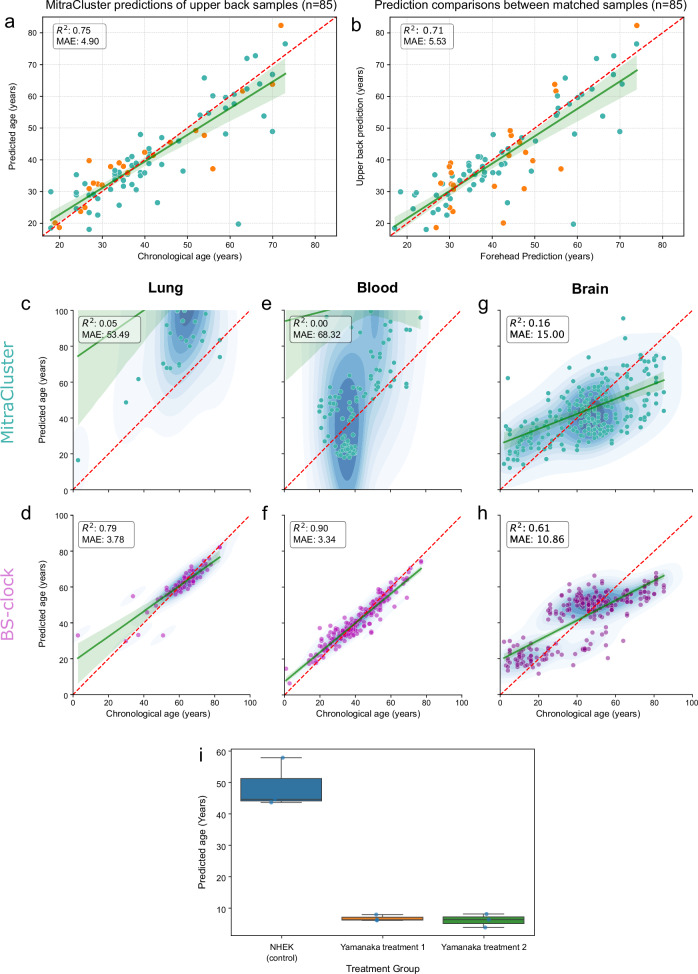
MitraCluster generalises across body sites, ectodermal tissues and captures rejuvenation. Spatial generalisability of MitraCluster was assessed using matched tape-strip samples from the face and upper back of 85 individuals. 61 out of 85 of these pairs shown here in teal have had the facial skin sample included in the training of the model, while the remaining 24 indicated in orange had not. MitraCluster accurately predicted age in upper back skin (all samples: *R*^2^ = 0.75, MAE = 4.90; teal only: *n* = 61, *R*^2^ = 0.71, MAE = 5.19; orange only:, *n* = 24, *R*^2^ = 0.84, MAE = 4.13) (**a**), with high concordance between predictions from both body sites (all samples: *R*^2^ = 0.71, MAE = 5.53; teal only: *n* = 61, *R*^2^ = 0.75, MAE = 4.93; orange only:, *n* = 24, *R*^2^ = 0.61, MAE = 7.04) (**b**), demonstrating reliable intra-individual consistency across sampling locations. Cross-tissue performance of MitraCluster (top row) and BS-Clock (bottom row) was evaluated using publicly available bisulfite sequencing datasets from lung (**c**, **d**), blood (**e**, **f**), and brain (**g**, **h**) tissue. For the BS-clock, the predictions presented correspond to the version specific to the corresponding tissue shown. BS-Clock performed well in blood and lung (*R*^2^ = 0.90 and 0.79; MAEs = 3.34 and 3.78), whereas MitraCluster underperformed in these tissues (*R*^2^ ≤ 0.05; MAEs > 50), consistent with its epidermis-specific training. However, in brain tissue, MitraCluster achieved moderate predictive performance (**g** MAE = 15.00, *R*^2^ = 0.16), approaching that of the brain-specific BS-Clock (**h** MAE = 10.86, *R*^2^ = 0.61). 59.1% of MitraCluster CpGs were covered on average by Blood samples, 75.2% by Lung and 83.7% by Brain samples. **i** Plot of age predictions from in vitro grown Normal Human Epidermal Keratinocytes under control conditions, or after two types of Yamanaka factor treatment supplementation that generate keratinocyte-derived iPSCs.

Given that our model was trained exclusively on facial skin, we evaluated the spatial generalisability of MitraCluster using matched tape-strip samples from the face and upper back of the same individual, from a total of 85 individuals. Of those, 61 of the facial skin samples participated in model training, while 24 were from participants not previously encountered by the model. These samples spanned a broad range of sequencing depths (2.95×–165×, mean = 17.82×) and were included without filtering to maximise representativeness. Despite lower coverage in the upper back (as low as 4×) and anatomical differences in exposure, MitraCluster maintained strong predictive accuracy (MAE = 4.90 years; *R*^2^ = 0.75) (Fig. 4a), nearly matching its performance on the independent facial skin validation set (MAE = 4.00 years). In addition, the prediction accuracy was comparable between upper-back samples from participants whose foreheads had been utilised in training (MAE = 5.19, *R*^2^ = 0.71) and those that had not (MAE = 4.13, *R*^2^ = 0.84) (Fig. 4a). By contrast, Horvath’s Skin & Blood clock showed substantially reduced accuracy on the same dataset (MAE = 10.76; *R*^2^ = 0.60), down from a MAE of 6.58 in facial skin (Supplementary Fig. 4a). The predicted ages between matched body sites were highly concordant, with a mean deviation of just 5.53 years, compared to 11.88 years for Horvath’s model (Fig. 4b and Supplementary Fig. 4b). Matched samples from participants not included in model training showed slightly reduced concordance to those included (*R*^2^ = 0.61, MAE = 7.04 and *R*^2^ = 0.75, MAE = 4.93, respectively) likely due to discrepancy in forehead prediction accuracy. Nonetheless, the overall strong Pearson correlation (*r* = 0.84) between paired samples suggests the model reliably captures a consistent skin aging signal across body sites.

We then assessed the broader applicability of MitraCluster on samples originating from tissues other than the skin, using a publicly available bisulfite sequencing dataset comprising brain, lung, and blood tissues also used by the BS-clock. MitraCluster underperformed for blood and lung samples (MAE = 68.32; *R*^2^ = 0 and MAE = 53.49; *R*^2^ = 0.05, respectively), which lie outside its training domain and are ontologically divergent tissues (Fig. 4c–f). However, it achieved better age prediction capacity in brain tissue (MAE = 15.00; *R*^2^ = 0.16), approaching that of the brain-specific BS-Clock (MAE = 10.86; *R*^2^ = 0.61), which was trained on brain data (Fig. 4g, h). This surprising result potentially suggests that MitraCluster captures an epigenetic aging programme conserved across ectoderm-derived tissues.

Having established the generalisability of our clock across tissues, sample types and processing methodologies, we attempted to assess its performance on in vitro material. We first applied it to a WGBS dataset from a primary keratinocyte cell line with known donor age^20,24^, and achieved reasonable accuracy with an absolute Δage value of 7.96 years (predicted age: 33.96, actual: 26). To assess this further, we then generated new EM-seq data from two commercially available keratinocyte cell lines derived from donors aged 21 and 52 years old. Material was collected at minimal passaging to minimise the described effect of population doubling on DNA methylation aging^5^. MitraCluster was able to distinguish between the younger and the older donor with reasonable accuracy, with predicted ages of 32.25 and 47.17 years, respectively (absolute Δage values of 11.54 and 4.83 years; Supplementary Table 4). Interestingly, the more accurate prediction corresponded to a cell line donated from eyelid tissue, which is anatomically proximal to the forehead, as opposed to the labial tissue where the younger sample was collected from. These results demonstrate that MitraCluster, despite being trained solely on in vivo facial epidermis, can capture age-associated signals that generalise to cells grown in culture.

This encouraging preliminary evidence for the in vitro generalisability of our models gave us the opportunity to evaluate their sensitivity to potential biological rejuvenation. To that end, we tested whether our models could detect age reversal in response to Yamanaka factor treatment, an established method for inducing stem cell-like states^25^. We identified a publicly available DNA methylation sequencing dataset from a parental primary keratinocyte cell line with and without two types of Yamanaka factor treatment, which generates keratinocyte-derived induced pluripotent stem-cells (iPSCs)^26^. These data were generated by WGBS at a shallow sequencing depth ranging between 1.5 and 3.5× and represented each treatment condition in triplicate. MitraCluster was able to capture a very substantial and consistent age reversal for all replicates subjected to both Yamanaka factor treatments tested (Fig. 4i). The average predicted age reduced from 48.8 years in differentiated control keratinocytes to 6.9 for iPSCs of treatment 1 and 6.2 for treatment 2 (Supplementary Table 4), described as more effective in producing naive iPSCs^26^. This finding, albeit limited, highlights the potential of our models to capture rejuvenation events even when those take place in in vitro settings where our models are not currently trained on.

## Discussion

Accurate and tissue-relevant biomarkers of biological aging are essential to advance both research and therapeutic development. In the context of skin, which is our largest, most environmentally exposed, and visibly aging organ, such tools, specifically for non-invasive assessment to allow mass use, are overdue. Skin aging is not only a cosmetic concern, but its health state influences the hallmarks of systemic aging^1^. To address this, the present study introduces the first epigenetic clocks trained on an EM-seq dataset of non-invasively collected human epidermis (*n* = 387). We evaluated two modelling strategies: MitraSolo, a single-CpG predictor, and MitraCluster, a region-based model that aggregates clusters of co-correlated CpGs. Both of our models exhibited state-of-the-art predictive capabilities for samples directly from the human epidermis, with accuracy surpassing established tools. The prediction accuracy was found to be sex-agnostic for both of our models and was not substantially affected by the Fitzpatrick score. MitraCluster achieved slightly higher predictive accuracy, likely due to its ability to buffer local sequencing noise and capture coordinated methylation patterns in biologically relevant regions. Unlike other clocks, such as Horvath’s PanTissue and Skin & Blood, PhenoAge or BS-clock, which were developed using blood, invasive biopsies or in vitro specimens, our models are directly trained on in vivo-derived keratinocyte-rich epidermal methylomes. This tissue specificity is important for capturing skin-relevant aging signatures.

We selected enzymatic methyl-sequencing (EM-seq) as the foundation for our database due to its ability to preserve DNA integrity and achieve broad methylome coverage—an essential feature for profiling low-input DNA from non-invasive tape-stripping of human skin^14,27,28^. Unlike arrays, which interrogate a limited set of predefined CpG sites, EM-seq captures millions of CpGs, including those in enhancer, shore, and intergenic regions often excluded from standard platforms^29^. This approach also allows exploration of DNA Methylation within the local context. Despite sequencing offering genome-wide potential, coverage can be uneven, and many array CpGs may fall below usable depth in sequencing datasets^13^. For example, BS-Clock filters out CpGs below 50× coverage but does not systematically assess the impact of depth on prediction accuracy.

To address this, we evaluated model performance across a range of sequencing depths. Both MitraSolo and MitraCluster maintained high accuracy at depths as low as 10×, despite being trained on data filtered at 15×. MitraCluster showed stable prediction performance across 10×–300× sequencing depth (MAE ≈ 4), suggesting that its region-based structure buffers against local noise. These results directly challenge the assumption that ultra-deep sequencing is necessary for reliable epigenetic clocks^9,19^ and establish a precedent for scalable, cost-effective biological age estimation in skin.

In benchmarking analyses, MitraCluster demonstrated better predictive performance (MAE = 4.00 years) compared to MitraSolo (MAE = 4.09), and outperformed array-based clocks like Horvath Skin & Blood (MAE = 6.58 years) and PhenoAge (MAE = 11.27 years) when applied to the same epidermal data. Most array-based clocks are trained on blood, explaining their low performance when evaluated on epidermal samples, but even the partially skin-trained Horvath Skin & Blood clock showed limited generalisability to our sample type. To allow for an even fairer cross-platform comparison, we decided to familiarise the well-established Horvath Pan-Tissue and Skin & Blood clocks with our data type. Retaining their original marker sets of CpGs, we re-trained both clocks using our training dataset and the same PCA-based approach we employed for our models. Surprisingly, the resulting models, and particularly the re-trained Skin & Blood clock, performed better than the originals, not only on our internal independent dataset but also on external WGBS skin data and even on a microarray epidermis dataset. Despite still not surpassing the Mitra models, the improved performance of the retrained microarray clocks advocates the value of our skin database in training superior clocks for skin aging.

Beyond outperforming these non-skin-specific clocks, MitraCluster also exceeded the accuracy of previously published skin-targeted models developed on array platforms. For example, clocks trained on 450 K/EPIC array data from suction blister samples, such as those by Brotmann et al. and Bienkowska et al., reported MAEs of approximately 6.4 years^30,31^. However, these models are constrained by the limited CpGs explored and the reduced sensitivity inherent to array technologies. As recently discussed by Gronniger et al., array-based clocks struggle with generalisability and often fail to capture subtle biological changes^2^. Unlike array-based models, MitraCluster leverages high-resolution sequencing from non-invasively collected epidermis, delivering superior accuracy, skin-specific relevance, and robustness across datasets. To rigorously test generalisability, we challenged the model with fully independent, publicly available datasets—despite three major obstacles: (i) most public resources rely on microarrays that lack coverage for many MitraCluster CpGs; (ii) available sequencing datasets often use different capture panels or suffer from low coverage; and (iii) most public skin samples are derived from biopsies rather than non-invasive methods. Nonetheless, we tested MitraCluster on an independent WGBS skin biopsy dataset (*n* = 19) to evaluate cross-platform performance. MitraCluster showed better accuracy (MAE = 6.52 years) in predicting the age of the samples compared to the WGBS-trained BS-Clock (MAE = 10.87 years)^13^. Taken together, these findings highlight the quality of our database and its appropriateness in the training of skin-specific epigenetic age predictors.

MitraCluster also demonstrated remarkable reproducibility in longitudinal skin sampling. Repeated measurements from the same individuals over time were more consistent than the model’s average prediction error, suggesting that age prediction deviations may reflect true biological variation rather than technical noise. This aligns with growing evidence that epigenetic age acceleration captures meaningful differences in tissue state, regenerative potential, and response to interventions^5,25,32^. This longitudinal robustness, coupled with MitraCluster’s capacity to capture biological responsiveness, establishes MitraCluster as a robust, biologically relevant tool for studying skin aging and quantifying treatment efficacy in clinical, cosmetic, and longevity-focused applications.

Moreover, MitraCluster demonstrated strong sampling location and tissue-level generalisability. When applied to matched facial skin and upper back tape-strip samples, predicted ages were highly concordant, indicating that the model captures a consistent aging signal across distinct skin sites. Small, systematic differences between locations may reflect local environmental exposures such as UV radiation or pollution, suggesting sensitivity to region-specific aging drivers. Further, MitraCluster showed good and coherent accuracy on an external bisulfite sequencing dataset from brain tissue (MAE = 15 years), approaching the brain-specific BS-clock performance (MAE = 10.86) trained specifically on that tissue and calculated with a Leave-One-Out approach. This performance highlights MitraCluster’s ability to generalise across ectoderm-derived tissues. Crucially, its success on bisulfite data, despite being trained on EM-seq, demonstrates methodological independence and supports its potential for future enrichment with external datasets and deployment across diverse platforms.

To assess the translational relevance of our models, we applied them to in vitro cultured keratinocyte cell lines. Despite challenges such as clonal purity and methylome changes from passaging, the model achieved strong predictive accuracy (absolute Δage values of 4.8–7.9 years), underscoring its robustness beyond in vivo contexts. We further tested the clocks’ sensitivity to biological rejuvenation using a publicly available dataset of epidermal keratinocytes reprogrammed into iPSCs via two types of Yamanaka factor treatment, an established model of age reversal^25,26,33^. Strikingly, MitraCluster successfully and reproducibly captured a substantial rejuvenating effect of the treatment, predicting the keratinocyte-derived iPSCs as approximately 42 years younger than the parental cell line. This supports the potential of the models to detect epigenetic rejuvenation in skin cells. As highlighted by Lujan et al.^34^, there is a growing demand for keratinocyte-specific methylation tools to evaluate therapeutic interventions, an unmet need addressed by our non-invasive, sequencing-based platform.

The skin-specific age predictors of this study represent a new class of epigenetic aging clocks: non-invasive, skin-specific, sequencing-native and region-aware. Their high accuracy, low-cost compatibility and practical relevance make them invaluable tools for both academic research and commercial skincare development. As skin health becomes a central focus of preventive aging and wellness, the skin-specific age predictors may offer a scalable solution to measure, monitor and modulate biological skin aging.

## Methods

### Sample population

The objectives of this study is fourfold: (i) to build a database for the development of robust and accurate sequencing-based DNA methylation clocks for human skin; (ii) to benchmark their performance against existing array- and bisulfite-based clocks; (iii) to evaluate their robustness across variable sequencing depths and biological replicates and (iv) to test their generalisability across body sites and other tissues.

The total dataset used in this study includes 817 skin samples collected by tape-stripping from 590 unique individuals between March 2021 and December 2024 at multiple sites across the UK and the US. Healthy volunteers aged 18–90 years with no significant concurrent illnesses or skin disease were included. All Fitzpatrick skin types and ethnicities were included. Volunteers who were taking any systemic or topical medication likely to interfere with the procedure were excluded, as were pregnant or breastfeeding women. A summary table of the volunteers stratified by sex, age bin and Fitzpatrick Score is included as Supplementary Table 3. Volunteers provided tape-stripping samples from facial skin, and a subset also provided matched upper back samples (*n* = 85). A subset of high-quality facial skin samples (*n* *=* 462) unique per individual was allocated for model training and independent testing. The remaining samples (*n* = 270) were not included in model training nor independent testing as biological replicates, non-facial-skin samples or due to low coverage. However, these samples were used for further evaluation, namely (i) robustness of the clock predictions to low coverage, (ii) assessment of within-individual prediction consistency across longitudinal biological replicates, (iii) anatomical generalisability and (iv) exploration of minimal sample quality in training. Volunteers shared demographic, environmental, medication, skin disease and personal history data.

All volunteer data has been collected in accordance with ethics and regulations. Volunteer recruitment was performed in different sites in the UK and the US. In the UK, data collection was approved by the Reading Independent Ethics Committee (RIEC 090221-2, 15 November 2020; RIEC 26032-1, 15 April 2021) and by the HRA and Health and Care Research Wales (REC 22/PR/0516, 30 May 2022). In the US, the study was approved by Advarra IRB, registered with OHRP and FDA under IRB#00000971 (IRB Pro00071502, 10 May 2023; IRB Pro00081364, 16 August 2024). The study was conducted in accordance with the ethical principles of the Declaration of Helsinki. All participants provided written informed consent prior to inclusion. Electronic data were anonymized and stored by Mitra Bio.

An additional set of publicly available 529 BS-seq samples from the DNA Methylation Haplotype Browser^35^ was used as an extra independent validation set, and to assess the generalisability of our models to other types of methylation conversion methods and tissues. This dataset is composed of 529 samples divided across four tissues: brain (*n* = 253), blood (*n* = 194), lung (*n* = 63) and skin (*n* = 19). DNA Methylation data for keratinocyte cell lines were produced in-house, as well as obtained from the following GEO accession numbers: GSM5652321^24^, GSE159297^26^. Details in Supplementary Table 4. A summary of how the data were used can be seen in Fig. 1.

All publicly available data have been listed in Supplementary Table 4.

### Sample collection and processing for sequencing

A total of eight consecutive large D-Squame tape strips (CuDerm, Dallas, TX, USA) of 3-cm diameter were collected from healthy skin areas on the face i.e. forehead. After cleansing with alcohol, each tape was applied to the skin twice, in the same sampling area, and pressure was applied for 5–10 s. The tape strips were subsequently transferred into a container with a DNA stabilising solution. Samples were stored at −20 °C until they were transported to the Mitra Bio laboratories (London, UK) on dry ice. There, genomic DNA was isolated from tape strips using a modified semi-automated version of the Quick-DNA HMW MagBead Kit protocol (ZymoResearch, Irvine, CA, USA) as previously described in Banila et al.^14^.

Libraries were prepared using a modified semi-automated version of NEBNext® Enzymatic Methyl-seq (EMseq) (New England Biolabs, Ipswich, MA, USA) according to the manufacturer’s instructions. Libraries were pooled equimolarly and subjected to hybridisation-based target enrichment using Twist Bioscience Human methylome panel (Twist Bioscience, San Francisco, CA, USA) and were quality controlled on a TapeStation 4150 HSD1000 (Agilent, Santa Clara, CA, USA). Sequencing was performed on Illumina NovaSeq 6000 and Novaseq X Plus flow cells (Illumina, San Diego, CA, USA).

### Primary data processing

The resulting FASTQ files were processed using an internal processing pipeline. Briefly, reads were trimmed with Trim Galore! version 0.6.6 (https://www.bioinformatics.babraham.ac.uk/projects/trim_galore/) and aligned to GRCh38.p13 using bwa-mem2 version 2.2.1 (https://github.com/bwa-mem2/bwa-mem2). The resulting BAM files were sorted and indexed using samtools version 1.10 (http://www.htslib.org), and methylated reads were calculated using MethylDackel after filtering them for at least 90% non-CpG C conversion to T using parameter --minConversionEfficiency 0.9 in module MethylDackel extract. (https://github.com/dpryan79/MethylDackel). This results in two matrices of dimensions 3,986,158×1 per sample, with rows corresponding to each individual CpG. One matrix indicates the number of fragments providing a read-out for the calculation, and the second matrix indicates the beta value of the CpG. For the Banila et al. data, matrices were subsetted to CpGs present in the capture panel. For the publicly available dataset, mhap tools^36^ was used to obtain the necessary coordinates from the available .mhap files and liftOver^37^ to match the genomic assembly of our dataset.

### Epigenetic clock model development and testing

For model training and validation, our total dataset of non-replicate, untreated, facial skin samples was subjected to initial filtering. Samples with an average sequencing depth of less than 15× and fewer than 90% of targeted CpGs covered were excluded. In turn, the number of available CpGs for feature selection was filtered by removing CpGs on chromosome X, Y and M, reducing available CpGs from 3,986,158 to 3,831,136. Then, the methylation values of individual CpGs with less than 7× sequencing depth were removed per sample. This process resulted in a dataset of 462 samples, which was divided into two subsets: a training set (*n* = 387, 83.76%) and an independent set (*n* = 75, 16.23%). We performed an initial reduction of the feature space by removing CpGs that tend to be poorly covered by removing CpGs missing in more than 70% of the samples. To avoid data leakage, we performed this filtering exclusively on the training set. This resulted in 3,830,117 available CpGs for feature selection. There is no overlap between the data cohorts used for testing and training. The age distribution of the testing dataset covers most of the range used in training.

We created two models, a single CpG model (MitraSolo) and a region-based model (MitraCluster). Both models were trained in Python using the Sci-Kit Learn library^38^. The models were trained on the same subset using the same sets for K-fold cross-validation. Missing values were imputed using the mean of each feature (CpG), and all features were standardised using *z*-score normalisation. Dimensionality reduction was performed in two steps: first, by selecting features based on their correlation with chronological age, and second, by applying principal component analysis (PCA) to further reduce feature dimensionality. Both models utilise PCA because it has been reported to enhance the robustness and accuracy of epigenetic clocks^29^, which is particularly relevant for longitudinal studies. For each fold in the outer cross-validation, the optimal hyperparameters for the Elastic Net linear regression models were selected using an inner cross-validation loop. The model outputs a transformed age value, using the original age transformation formula defined by Horvath et al.^39^, with the adult age threshold set at 20 years. To develop the region-based model (MitraCluster), we first identified genomic regions comprising clusters of CpGs that exhibit coordinated correlation with chronological age in the Mitra Bio database. CpGs were grouped into regions based on their sequential proximity and similarity in age-correlation patterns. Specifically, adjacent CpGs were merged if the absolute difference in correlation coefficients, either relative to the region’s initial CpG or between consecutive sites, remained below a defined tolerance threshold selected as described below. For each resulting region, the mean beta value was calculated and used as the input feature in model training, providing a smoothed and biologically interpretable representation of age-associated methylation patterns. Multiple values of this parameter were tested, and a tolerance of 0.075 was selected. Only regions with at least 3 CpGs in their span were considered for this approach. A total of 245,198 regions were included.

### Training of sequencing-based models using biomarkers from microarray clocks

We evaluated the performance of microarray-derived biomarkers by training two extra models on the Mitra training dataset after enforcing the selection of CpGs to match those used by established microarray clocks. We created two models, the first one based on Horvath’s Pan-Tissue clock^20^, and the second based on Horvath’s Skin and Blood clock^39^. To do so, we retrieved the specific Illumina CpG IDs from MethylCypher^40^. The CpG IDs were matched to the human genome hg38 and the corresponding Twist CpGs overlapping these regions were identified. Out of the 353 CpGs used for Horvath’s Pan-Tissue, our data overlaps 346 of them, representing 98% of the necessary clock CpGs. In the case of Horvath’s Skin and Blood CpGs, our data includes 387 out of 391 (99%). The models were trained using the same PCA-based approach, hyperparameters and K-fold cross-validation as MitraSolo, and features subsetted only to those identified by the previous overlaps. When these clocks are used on microarray data, the CpG values of the microarray datasets are translated from microarray to CpG-coordinate format in the same way as defined above.

### Genomic feature overlap assessment

Clock CpGs were intersected with genomic feature intervals to identify overlaps across the genome using the R package annotatR and internal genomic feature and CpG context interval files^41^. To identify if these overlaps occurred more often than expected at random for the various genomic features, we developed a Monte-Carlo-based enrichment assessment to estimate the statistical significance of these overlaps. We used the R package regioneR^42^ to obtain randomised sets of CpGs of equal size and similar chromosomal distribution to those used by each of the clocks, from the available training set. We then intersect those with the genomic annotations interval files, as we performed for the actual clock CpGs and calculate counts of overlaps. Per CpG, overlaps with each type of annotation are counted at most once. We repeat this process of random generation, overlapping and calculation 250 times, producing a background distribution of overlaps per genomic feature. We consider this distribution normal for all annotation types, and we calculate the significance of enrichment by performing a two-tailed single-sample t-test comparison between actual and simulated counts of overlap.

### Gene annotation and pathway enrichment analysis

Overlapping CpGs between MitraSolo and MitraCluster were identified and assigned to the gene with the most proximal transcriptional start site using ChIPSeeker^43^. The resulting gene list was used for pathway enrichment analysis using the ‘enrichr’ function from the ‘gseapy’^44^ Python package, using as background all possible genes assigned to the target panel. Five sources were used for the gene sets: KEGG^45^, reactome^46^ and the three gene ontology^47^ trees; biological process, molecular function and cellular compartment.

### Statistical analysis

We assessed the predictive performance of our model using five standard regression metrics: MAE, MSE, RMSE, MAD and the *R*^2^. These metrics quantify the agreement between predicted and actual chronological age. MAE provides an intuitive measure of average prediction error, allowing straightforward comparisons across models. However, because MAE is sensitive to outliers, MAD was also included to capture the typical magnitude of error in a more robust manner, offering a better representation of central performance when error distributions are skewed or contain extreme values. Throughout our study, we also report Δage, which we define as the difference between actual chronological and clock-predicted age as a measure to assess prediction accuracy.

### Use and transformation of external models and datasets

To benchmark our sequencing-derived methylation profiles against established microarray-based epigenetic clocks, we applied four widely used clocks, Horvath’s Pan-Tissue^20^, Horvath’s Skin & Blood^15^, Hannum^21^ and PhenoAge^22^, to an independent test dataset (*n* = 75). For each clock, CpG probes required by the original models were matched to our sequencing data. This approach enabled coverage of at least 93% of the CpGs used by the microarray clocks (Table 1). Predictions for each clock were performed using the R package MethylCIPHER^40^. Additionally, we predicted the epigenetic ages of our data using a sequencing-based model, BS-Clock, available at https://github.com/hucongcong97/BS-clock/tree/main. Although BS-Clock models were trained separately on four tissue types, only the blood-trained model is publicly available and was used in our analyses on our independent test set. To ensure compatibility, we converted our sample coordinates from hg38 to hg19. Of the 4527 CpGs used in BS-Clock, at least 52.97% (2398 sites) were missing per sample. To maximise overlap, we attempted nearest-CpG substitution for missing sites within a 2000 bp window, which marginally increased feature coverage but failed to improve predictive accuracy. For benchmarking of our clocks on Bisulfite-sequencing data, BS-Clock age estimates from its original repository—calculated using tissue-matched models- were used to evaluate performance on their own dataset, ensuring fair comparison against MitraCluster predictions. To perform a more meaningful comparison between the microarray clocks and their MitraSolo counterparts, we obtained a publicly available dataset (*n* = 28) of DNA Methylation in isolated epidermis^23^, measured with Illumina’s Infinium HumanMethylation450 BeadChip. The probe IDs were translated into hg38 coordinates using an hg38 Illumina manifest (https://github.com/zhou-lab/InfiniumAnnotationV1/raw/main/HM450/homo_sapiens.tsv.gz).

**Table 1:** Total number of CpGs per clock and the subset directly overlapping with our sequencing data

Clock	Type	Total probes	Probes present	Percent present
PhenoAge	Microarray	513	510	99%
Hannum	Microarray	71	66	93%
Horvath1	Microarray	353	346	98%
Horvath2	Microarray	391	387	99%
BS-Clock	Sequencing	4527	2129	47%

Microarray clocks have a minimum percentage of probe representation of 93%, whilst the Twist panel only has a direct overlap to 47% of the BS-Clock probes.

## Supplementary information

Supplementary Information

## Data Availability

The data that support the findings of this study are available from Mitra Bio, but restrictions apply to the availability of these data, which were used under licence for the current study, and so are not publicly available. Data are, however, available from the authors upon reasonable request and with permission of Mitra Bio.

## Code availability

The underlying code for this study and training/validation datasets is not publicly available but may be made available to qualified researchers on reasonable request from the corresponding author.
