# Supplementary material for: Epigenetic age predictors for non-invasive assessment of human skin
Source: NPJ Aging. 2025 Dec 12;12(1):11. doi: 10.1038/s41514-025-00314-0 (PMC12820032; doi:10.1038/s41514-025-00314-0)
Supplement: Supplementary file 1 — Supplementary Information [file 41514_2025_314_MOESM1_ESM.pdf]

Supplementary Figures and Tables

Epigenetic age predictors for non-invasive assessment of human skin

Angel Menendez Vazquez[1], Dimitris Katsanos[1], Miruna Vasile[1], Andrew Graham[1], Victoria Dyster[1], Shakiba Kaveh[1], Mahdi Moqri[2], Cristiana Banila[1]

[1]Mitra Bio, Translation and Innovation Hub, London, UK
[2]Division of Genetics, Department of Medicine, Brigham and Women's Hospital, Harvard Medical School, Boston, MA, USA

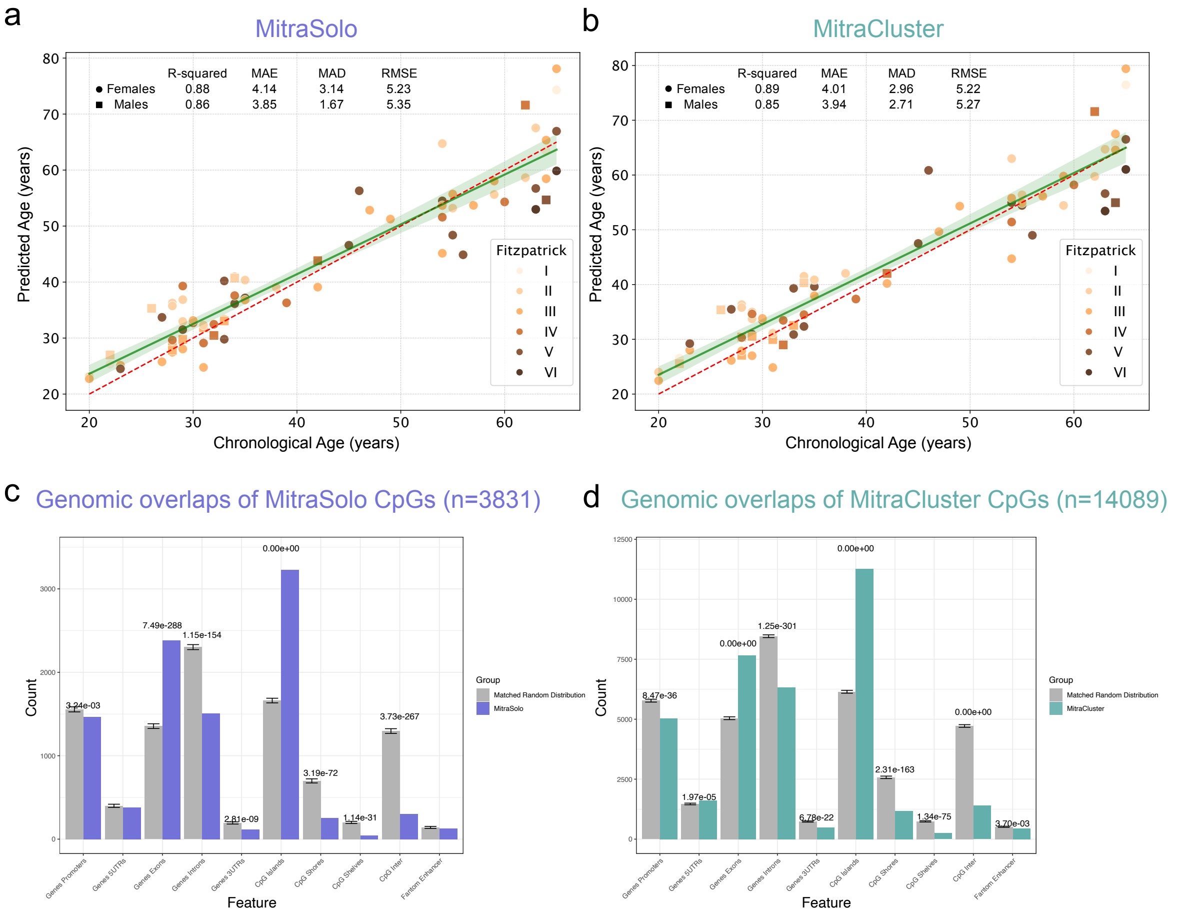

Supplemental Figure 1. Mitra clocks are sex-agnostic and their CpGs are enriched in coding sequences. a-b) Regression plots of predicted age to chronological for the MitraSolo a) and the MitraCluster b) clocks using the independent validation dataset. Circles indicate females and squares males and the sex-specific clock accuracy metrics are presented at the top of each plot. Points are coloured according to the participant Fitzpatrick score (performance metrics Supplemental Table 1). **c-d)** Barplots of counts of overlaps identified between the CpGs used by MitraSolo c) and MitraCluster d) and various genomic features and CpG contexts. Bars in colour are the actual counts of overlaps between a particular type of feature and CpGs while bars in grey represent the average overlap within the error rate calculated from multiple simulations of randomised CpG localisation within the workable genome. Error bars represent the standard deviation of the mean simulated overlap. P values are shown above the bar when there is statistically significant enrichment or depletion of overlap with any particular feature.

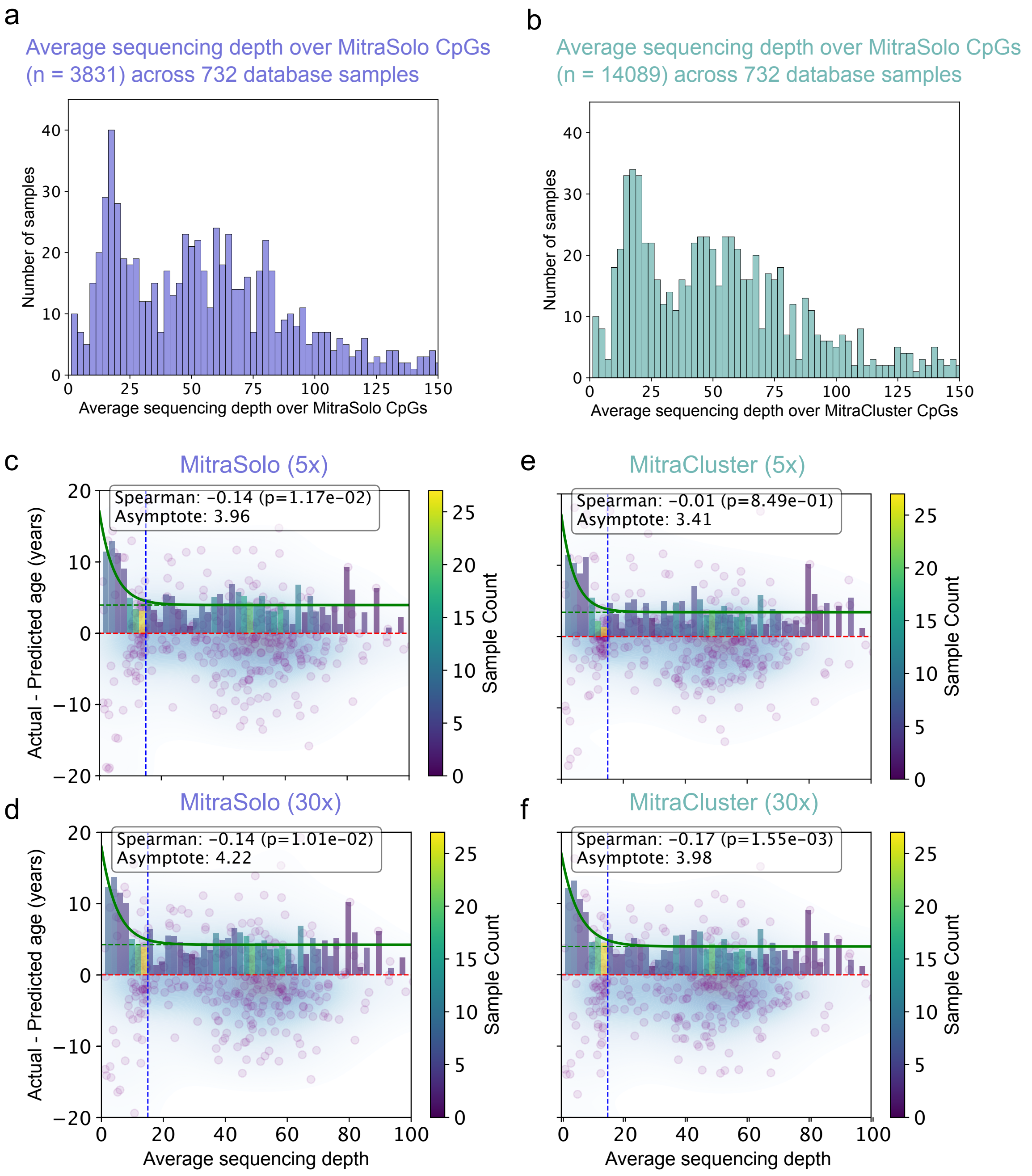

Supplemental Figure 2. Database variation in average clock sequencing depth and the effect of sequencing depth thresholds during training on prediction accuracy for Mitra models. a-b) Histograms of the count of forehead tape-stripping database samples (n=732), at different bins of average sequencing depth, for the 3831 CpGs utilised by the MitraSolo clock a) and the 14089 CpGs of the MitraCluster. **c-f)** Actual chronological age minus predicted age is plotted against average CpG sequencing depth for the single-CpG MitraSolo (c–d) and the region-based MitraCluster (e–f) under different minimum sequencing depth thresholds used during model training: 5× (c, e), and 30× (d, f). In each panel, the Δage values are shown as density-coloured scatter plots, and accompanying histograms display sample count distributions across coverage bins. The blue vertical line indicates a coverage of 15×, and an asymptotic trendline (green) illustrates saturation of prediction error with increasing depth. Spearman correlation statistics are included at the top of each plot. Across all conditions, both models exhibit stable Δage values distributions at coverage ≥10×, with asymptotic MAEs near ~4 years, confirming that shallow sequencing does not substantially impair model performance.

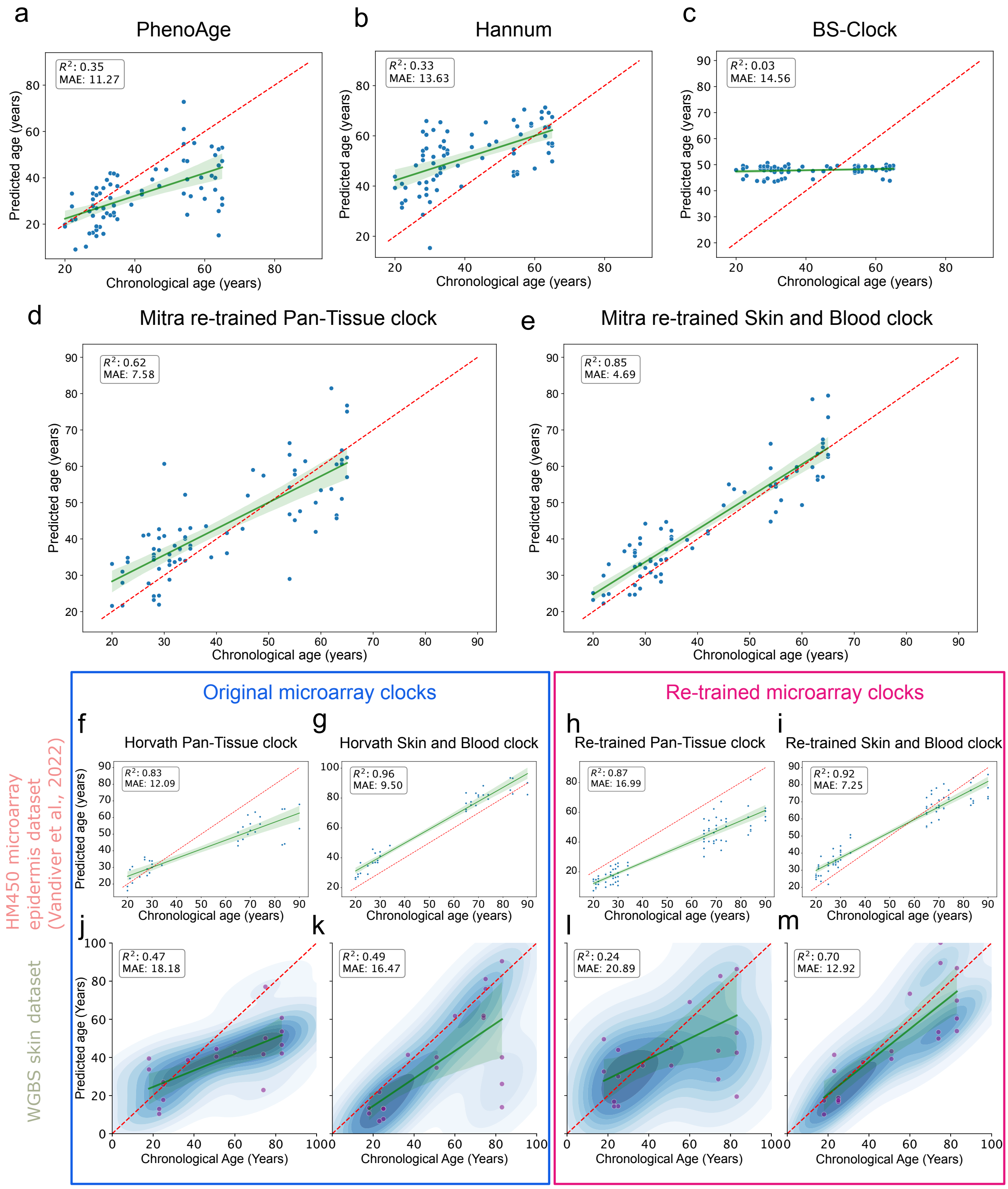

Supplemental Figure 3. Comparative performance of established epigenetic clocks and re-trained clocks sequencing and microarray datasets. a-c) Predicted age versus chronological age is shown for the independent test set (n = 75) across all assessed models. Panels a-b show predictions from widely used array-based clocks: PhenoAge (a), and Hannum (b). These clocks, all trained on blood or biopsy tissue, exhibit substantially higher prediction errors (e.g., MAE = 13.63 for Hannum) when applied to tape-stripped skin DNA. Panel (c) shows predictions from the BS-Clock model, trained on high-resolution bisulfite sequencing data from blood, which fails to generalize to epidermal data. **d-e)** Regression plots of predicted age to chronological age for the independent validation dataset using the re-trained versions of the established Horvath's microarray Pan-Tissue d) and Skin & Blood clocks e) that use the same CpGs for prediction to the originals but have been trained on the Mitra training dataset. **f-m)** Regression plots of predicted age to chronological age for a

microarray dataset of human epidermis samples f-i) and a WGBS dataset of human skin using the original f-g, j-k) or the re-trained h-i, l-m) Pan-Tissue and Skin & Blood clocks.

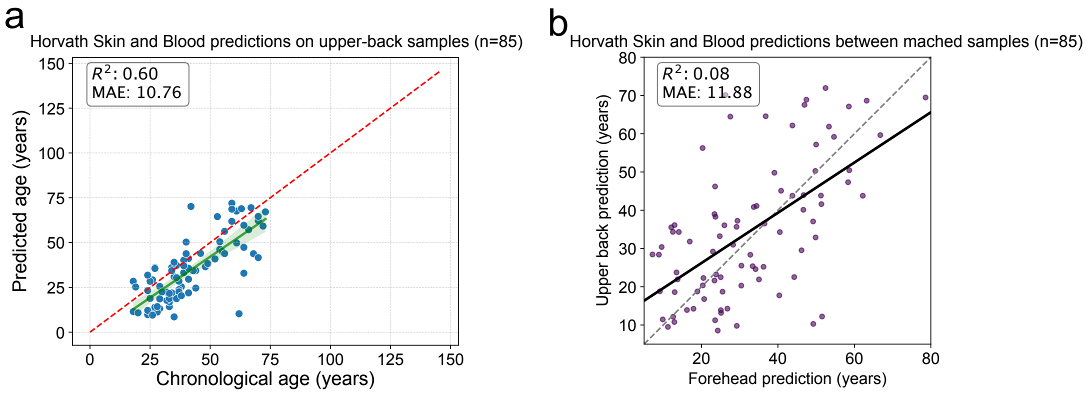

Supplemental Figure 4. Forehead and Upper back prediction comparisons by Horvath Skin and Blood clock a–b). Spatial generalizability of the Horvath Skin and Blood clock was assessed using matched tape-strip samples from the forehead and upper back of 85 individuals. Horvath Skin and Blood clock predicted age in upper back skin with lower accuracy (R^2 = 0.60, MAE = 10.76) (a), The concordance between the two sampling locations was suboptimal (R^2 = 0.08, MAE = 11.88) (b).

Supplementary tables

Supplemental Table 1: Prediction metrics in the independent dataset by clock, subsetted by the Fitzpatrick score of the individual. This table also includes information about the number of individuals by sex in each group. Predictions do not seem to be affected by Fitzpatrick score.

Clock	Fitzpatrick	Dataset	n_females	n_males	R2	MAE	MAD	RMSE
MitraCluster	All	independent	63	12	0.89	4.00	2.96	5.23
MitraCluster	I	independent	3	0	0.96	5.84	4.56	7.18
MitraCluster	II	independent	15	3	0.92	4.84	4.65	5.60
MitraCluster	III	independent	20	5	0.93	3.00	1.99	4.36
MitraCluster	IV	independent	9	3	0.92	2.70	2.06	3.71
MitraCluster	V	independent	14	1	0.81	4.95	4.62	6.24
MitraCluster	VI	independent	2	0	1.00	6.78	6.78	7.33
MitraSolo	All	independent	63	12	0.88	4.09	3.14	5.25
MitraSolo	I	independent	3	0	0.96	4.42	3.35	5.72
MitraSolo	II	independent	15	3	0.91	5.04	4.75	5.84
MitraSolo	III	independent	20	5	0.93	2.81	1.79	4.11
MitraSolo	IV	independent	9	3	0.86	3.70	2.56	4.80
MitraSolo	V	independent	14	1	0.83	4.88	3.21	5.95
MitraSolo	VI	independent	2	0	1.00	7.58	7.58	7.96

Supplemental Table 2: List of significantly enriched pathways is presented in the table below.

Gene_set	Term	P-value	Adjusted P-value	-log(FDR)	Odds Ratio	Combined Score
GO_Molecular_Function_2021	RNA polymerase II transcription regulatory region sequence-specific DNA binding (GO:0000977)	5.6E-22	1.7E-19	4.3E+01	4.4E+00	2.2E+02
GO_Biological_Process_2021	regulation of transcription by RNA polymerase II (GO:0006357)	3.8E-18	6.3E-15	3.3E+01	3.3E+00	1.3E+02
GO_Molecular_Function_2021	sequence-specific DNA binding (GO:0043565)	4.6E-17	6.9E-15	3.3E+01	5.1E+00	1.9E+02
GO_Molecular_Function_2021	double-stranded DNA binding (GO:0003690)	1.4E-15	1.0E-13	3.0E+01	5.0E+00	1.7E+02
GO_Molecular_Function_2021	RNA polymerase II cis-regulatory region sequence-specific DNA binding (GO:0000978)	1.3E-15	1.0E-13	3.0E+01	3.9E+00	1.3E+02
GO_Molecular_Function_2021	cis-regulatory region sequence-specific DNA binding (GO:0000987)	5.1E-15	3.0E-13	2.9E+01	3.8E+00	1.2E+02
GO_Biological_Process_2021	regulation of transcription, DNA-templated (GO:0006355)	9.4E-16	7.9E-13	2.8E+01	3.0E+00	1.1E+02
GO_Molecular_Function_2021	sequence-specific double-stranded DNA binding (GO:1990837)	3.0E-14	1.5E-12	2.7E+01	4.6E+00	1.4E+02
GO_Molecular_Function_2021	transcription cis-regulatory region binding (GO:0000976)	1.6E-08	6.7E-07	1.4E+01	3.8E+00	6.8E+01
KEGG_2021_Human	Neuroactive ligand-receptor interaction	8.5E-09	1.7E-06	1.3E+01	4.8E+00	9.0E+01
GO_Biological_Process_2021	nervous system development (GO:0007399)	1.5E-08	7.4E-06	1.2E+01	4.2E+00	7.5E+01
GO_Biological_Process_2021	excitatory chemical synaptic transmission (GO:0098976)	1.8E-08	7.4E-06	1.2E+01	1.6E+02	2.9E+03
GO_Cellular_Component_2021	integral component of plasma membrane (GO:0005887)	5.0E-08	9.1E-06	1.2E+01	2.5E+00	4.3E+01
Reactome_2022	Signaling By GPCR R-HSA-372790	2.0E-07	1.1E-04	9.1E+00	3.2E+00	4.9E+01
GO_Molecular_Function_2021	neuropeptide receptor activity (GO:0008188)	3.7E-06	1.2E-04	9.0E+00	1.8E+01	2.2E+02
GO_Molecular_Function_2021	G protein-coupled receptor activity (GO:0004930)	3.5E-06	1.2E-04	9.0E+00	4.3E+00	5.4E+01
Reactome_2022	GPCR Ligand Binding R-HSA-500792	4.7E-07	1.4E-04	8.9E+00	3.7E+00	5.3E+01
Reactome_2022	GPCR Downstream Signaling R-HSA-388396	7.4E-07	1.4E-04	8.9E+00	3.2E+00	4.5E+01
GO_Cellular_Component_2021	neuron projection (GO:0043005)	3.2E-06	2.9E-04	8.2E+00	3.1E+00	4.0E+01
GO_Cellular_Component_2021	ionotropic glutamate receptor complex (GO:0008328)	7.0E-06	4.2E-04	7.8E+00	1.5E+01	1.8E+02
KEGG_2021_Human	cAMP signaling pathway	5.5E-06	4.8E-04	7.6E+00	4.7E+00	5.7E+01
KEGG_2021_Human	Cocaine addiction	7.4E-06	4.8E-04	7.6E+00	1.1E+01	1.3E+02
Reactome_2022	G Alpha (I) Signaling Events R-HSA-418594	5.7E-06	8.2E-04	7.1E+00	3.9E+00	4.7E+01
GO_Molecular_Function_2021	glutamate-gated calcium ion channel activity (GO:0022849)	3.6E-05	1.1E-03	6.8E+00	9.6E+01	9.9E+02
GO_Biological_Process_2021	positive regulation of transcription by RNA polymerase II (GO:0045944)	5.9E-06	2.0E-03	6.2E+00	2.6E+00	3.1E+01
GO_Biological_Process_2021	positive regulation of transcription, DNA-templated (GO:0045893)	7.7E-06	2.0E-03	6.2E+00	2.3E+00	2.7E+01
GO_Biological_Process_2021	central nervous system development (GO:0007417)	8.3E-06	2.0E-03	6.2E+00	4.2E+00	4.9E+01
GO_Molecular_Function_2021	ligand-gated channel activity (GO:0022834)	7.5E-05	2.0E-03	6.2E+00	1.3E+01	1.3E+02
GO_Molecular_Function_2021	ligand-gated ion channel activity (GO:0015276)	8.9E-05	2.0E-03	6.2E+00	1.3E+01	1.2E+02
GO_Molecular_Function_2021	ionotropic glutamate receptor activity (GO:0004970)	8.8E-05	2.0E-03	6.2E+00	2.1E+01	2.0E+02
GO_Cellular_Component_2021	postsynaptic density (GO:0014069)	4.6E-05	2.1E-03	6.2E+00	5.2E+00	5.2E+01
GO_Molecular_Function_2021	voltage-gated cation channel activity (GO:0022843)	1.1E-04	2.3E-03	6.1E+00	6.0E+00	5.5E+01
KEGG_2021_Human	Glutamatergic synapse	6.7E-05	3.3E-03	5.7E+00	5.6E+00	5.4E+01
Reactome_2022	Class A/1 (Rhodopsin-like Receptors) R-HSA-373076	3.1E-05	3.6E-03	5.6E+00	3.6E+00	3.7E+01
GO_Molecular_Function_2021	NMDA glutamate receptor activity (GO:0004972)	1.9E-04	3.8E-03	5.6E+00	3.8E+01	3.3E+02
GO_Cellular_Component_2021	cation channel complex (GO:0034703)	1.1E-04	4.1E-03	5.5E+00	7.1E+00	6.4E+01
KEGG_2021_Human	Circadian entrainment	1.2E-04	4.8E-03	5.3E+00	5.9E+00	5.3E+01
GO_Biological_Process_2021	regulation of neurotransmitter receptor activity (GO:0099601)	2.4E-05	5.1E-03	5.3E+00	9.2E+00	9.8E+01

**GO_Molecular_Function_2021**	neuropeptide binding (GO:0042923)	2.7E-04	5.1E-03	5.3E+00	1.5E+01	1.2E+02
**GO_Biological_Process_2021**	chemical synaptic transmission, postsynaptic (GO:0099565)	2.9E-05	5.3E-03	5.2E+00	1.7E+01	1.8E+02
**GO_Cellular_Component_2021**	NMDA selective glutamate receptor complex (GO:0017146)	1.9E-04	5.8E-03	5.2E+00	3.8E+01	3.3E+02
**GO_Cellular_Component_2021**	excitatory synapse (GO:0060076)	2.2E-04	5.8E-03	5.2E+00	1.6E+01	1.4E+02
**Reactome_2022**	Adrenaline,noradrenaline Inhibits Insulin Secretion R-HSA-400042	6.3E-05	6.0E-03	5.1E+00	1.4E+01	1.4E+02
**KEGG_2021_Human**	Nicotine addiction	2.2E-04	6.8E-03	5.0E+00	1.0E+01	8.8E+01
**KEGG_2021_Human**	Calcium signaling pathway	2.4E-04	6.8E-03	5.0E+00	3.6E+00	3.0E+01
**GO_Molecular_Function_2021**	DNA binding (GO:0003677)	3.9E-04	6.9E-03	5.0E+00	2.2E+00	1.7E+01
**GO_Biological_Process_2021**	neuron differentiation (GO:0030182)	5.8E-05	7.7E-03	4.9E+00	4.6E+00	4.5E+01
**GO_Biological_Process_2021**	brain development (GO:0007420)	6.0E-05	7.7E-03	4.9E+00	5.1E+00	4.9E+01
**GO_Biological_Process_2021**	chemical synaptic transmission (GO:0007268)	5.2E-05	7.7E-03	4.9E+00	3.6E+00	3.5E+01
**GO_Biological_Process_2021**	G protein-coupled receptor signaling pathway, coupled to cyclic nucleotide second messenger (GO:0007187)	5.3E-05	7.7E-03	4.9E+00	8.1E+00	8.0E+01
**GO_Molecular_Function_2021**	DNA-binding transcription activator activity, RNA polymerase II-specific (GO:0001228)	5.2E-04	8.6E-03	4.8E+00	3.0E+00	2.2E+01
**Reactome_2022**	G Alpha (S) Signaling Events R-HSA-418555	1.3E-04	1.1E-02	4.5E+00	4.6E+00	4.1E+01
**GO_Molecular_Function_2021**	nucleotide diphosphatase activity (GO:0004551)	7.3E-04	1.1E-02	4.5E+00	2.1E+01	1.5E+02
**GO_Biological_Process_2021**	negative regulation of catecholamine secretion (GO:0033604)	1.2E-04	1.3E-02	4.4E+00	4.8E+01	4.3E+02
**GO_Biological_Process_2021**	regulation of postsynaptic membrane potential (GO:0060078)	1.2E-04	1.3E-02	4.4E+00	1.2E+01	1.1E+02
**GO_Biological_Process_2021**	positive regulation of gastrulation (GO:2000543)	1.2E-04	1.3E-02	4.4E+00	4.8E+01	4.3E+02
**Reactome_2022**	ADORA2B Mediated Anti-Inflammatory Cytokine Production R-HSA-9660821	1.8E-04	1.3E-02	4.3E+00	4.9E+00	4.2E+01
**Reactome_2022**	Unblocking Of NMDA Receptors, Glutamate Binding And Activation R-HSA-438066	2.2E-04	1.4E-02	4.3E+00	1.6E+01	1.4E+02
**GO_Biological_Process_2021**	anterior/posterior axis specification (GO:0009948)	1.4E-04	1.4E-02	4.2E+00	1.8E+01	1.6E+02
**Reactome_2022**	Amine Ligand-Binding Receptors R-HSA-375280	2.8E-04	1.5E-02	4.2E+00	9.8E+00	8.0E+01
**Reactome_2022**	Adrenoceptors R-HSA-390696	2.9E-04	1.5E-02	4.2E+00	3.2E+01	2.6E+02
**KEGG_2021_Human**	Maturity onset diabetes of the young	6.4E-04	1.6E-02	4.2E+00	1.2E+01	8.6E+01
**GO_Biological_Process_2021**	regulation of insulin secretion (GO:0050796)	1.8E-04	1.6E-02	4.1E+00	5.6E+00	4.8E+01
**GO_Biological_Process_2021**	excitatory postsynaptic potential (GO:0060079)	1.8E-04	1.6E-02	4.1E+00	1.7E+01	1.5E+02
**Reactome_2022**	Long-term Potentiation R-HSA-9620244	3.9E-04	1.9E-02	4.0E+00	1.4E+01	1.1E+02
**GO_Molecular_Function_2021**	G protein-coupled peptide receptor activity (GO:0008528)	1.4E-03	2.1E-02	3.9E+00	5.3E+00	3.5E+01
**GO_Cellular_Component_2021**	nucleus (GO:0005634)	9.6E-04	2.2E-02	3.8E+00	1.5E+00	1.0E+01
**GO_Biological_Process_2021**	cellular response to estradiol stimulus (GO:0071392)	2.7E-04	2.2E-02	3.8E+00	1.5E+01	1.2E+02
**GO_Biological_Process_2021**	long-term synaptic potentiation (GO:0060291)	2.7E-04	2.2E-02	3.8E+00	1.5E+01	1.2E+02
**GO_Molecular_Function_2021**	ion channel activity (GO:0005216)	1.8E-03	2.5E-02	3.7E+00	5.0E+00	3.2E+01
**GO_Molecular_Function_2021**	adrenergic receptor binding (GO:0031690)	1.8E-03	2.5E-02	3.7E+00	1.5E+01	9.4E+01
**GO_Biological_Process_2021**	regulation of cation channel activity (GO:2001257)	3.3E-04	2.5E-02	3.7E+00	5.9E+00	4.7E+01
**GO_Biological_Process_2021**	neuropeptide signaling pathway (GO:0007218)	3.6E-04	2.6E-02	3.7E+00	7.0E+00	5.6E+01
**GO_Biological_Process_2021**	regulation of nervous system development (GO:0051960)	4.1E-04	2.6E-02	3.6E+00	8.9E+00	7.0E+01
**GO_Biological_Process_2021**	negative regulation of transcription, DNA-templated (GO:0045892)	3.9E-04	2.6E-02	3.6E+00	2.1E+00	1.7E+01
**GO_Biological_Process_2021**	positive regulation of developmental process (GO:0051094)	3.8E-04	2.6E-02	3.6E+00	4.0E+00	3.1E+01
**GO_Molecular_Function_2021**	alpha-2A adrenergic receptor binding (GO:0031694)	2.3E-03	2.7E-02	3.6E+00	4.3E+01	2.6E+02

**GO_Molecular_Function_2021**	LBD domain binding (GO:0050693)	2.3E-03	2.7E-02	3.6E+00	4.3E+01	2.6E+02
**GO_Molecular_Function_2021**	ankyrin repeat binding (GO:0071532)	2.3E-03	2.7E-02	3.6E+00	4.3E+01	2.6E+02
**GO_Biological_Process_2021**	regulation of AMPA receptor activity (GO:2000311)	4.7E-04	2.9E-02	3.5E+00	1.3E+01	9.9E+01
**Reactome_2022**	G Alpha (Z) Signaling Events R-HSA-418597	7.7E-04	3.2E-02	3.5E+00	7.7E+00	5.5E+01
**Reactome_2022**	Peptide Ligand-Binding Receptors R-HSA-375276	7.3E-04	3.2E-02	3.5E+00	3.6E+00	2.6E+01
**GO_Biological_Process_2021**	metanephric mesenchyme development (GO:0072075)	5.5E-04	3.2E-02	3.4E+00	2.4E+01	1.8E+02
**GO_Biological_Process_2021**	metanephros development (GO:0001656)	5.5E-04	3.2E-02	3.4E+00	1.2E+01	9.2E+01
**GO_Molecular_Function_2021**	ligand-gated calcium channel activity (GO:0099604)	3.0E-03	3.3E-02	3.4E+00	1.2E+01	7.0E+01
**GO_Molecular_Function_2021**	glutamate receptor activity (GO:0008066)	3.0E-03	3.3E-02	3.4E+00	1.2E+01	7.0E+01
**GO_Biological_Process_2021**	kidney development (GO:0001822)	5.9E-04	3.3E-02	3.4E+00	6.3E+00	4.7E+01
**GO_Molecular_Function_2021**	dihydropyrimidinase activity (GO:0004157)	3.4E-03	3.4E-02	3.4E+00	3.2E+01	1.8E+02
**GO_Molecular_Function_2021**	epinephrine binding (GO:0051379)	3.4E-03	3.4E-02	3.4E+00	3.2E+01	1.8E+02
**GO_Molecular_Function_2021**	alpha-adrenergic receptor activity (GO:0004936)	3.4E-03	3.4E-02	3.4E+00	3.2E+01	1.8E+02
**GO_Biological_Process_2021**	regulation of insulin secretion involved in cellular response to glucose stimulus (GO:0061178)	6.4E-04	3.5E-02	3.4E+00	1.2E+01	8.6E+01
**Reactome_2022**	Anti-inflammatory Response Favoring Leishmania Infection R-HSA-9662851	9.4E-04	3.6E-02	3.3E+00	3.8E+00	2.7E+01
**GO_Biological_Process_2021**	positive regulation of transporter activity (GO:0032411)	7.3E-04	3.7E-02	3.3E+00	2.1E+01	1.5E+02
**GO_Biological_Process_2021**	heart development (GO:0007507)	7.5E-04	3.7E-02	3.3E+00	4.0E+00	2.9E+01
**GO_Biological_Process_2021**	urogenital system development (GO:0001655)	7.3E-04	3.7E-02	3.3E+00	2.1E+01	1.5E+02
**GO_Biological_Process_2021**	generation of neurons (GO:0048699)	7.9E-04	3.8E-02	3.3E+00	3.6E+00	2.6E+01
**GO_Biological_Process_2021**	positive regulation of synaptic transmission (GO:0050806)	8.1E-04	3.8E-02	3.3E+00	6.0E+00	4.2E+01
**GO_Biological_Process_2021**	vasodilation (GO:0042311)	9.3E-04	4.1E-02	3.2E+00	1.9E+01	1.3E+02
**GO_Biological_Process_2021**	pancreas development (GO:0031016)	9.3E-04	4.1E-02	3.2E+00	1.9E+01	1.3E+02
**Reactome_2022**	Regulation Of Insulin Secretion R-HSA-422356	1.2E-03	4.1E-02	3.2E+00	5.5E+00	3.7E+01
**GO_Molecular_Function_2021**	mRNA 3'-UTR AU-rich region binding (GO:0035925)	4.5E-03	4.3E-02	3.1E+00	1.0E+01	5.5E+01
**GO_Cellular_Component_2021**	neurofibrillary tangle (GO:0097418)	2.3E-03	4.6E-02	3.1E+00	4.3E+01	2.6E+02
**GO_Biological_Process_2021**	regulation of NMDA receptor activity (GO:2000310)	1.1E-03	4.8E-02	3.0E+00	9.9E+00	6.7E+01
**GO_Biological_Process_2021**	regulation of gastrulation (GO:0010470)	1.2E-03	4.8E-02	3.0E+00	1.7E+01	1.2E+02
**GO_Biological_Process_2021**	genitalia development (GO:0048806)	1.2E-03	4.8E-02	3.0E+00	1.7E+01	1.2E+02
**GO_Biological_Process_2021**	regulation of cell population proliferation (GO:0042127)	1.2E-03	4.8E-02	3.0E+00	2.1E+00	1.4E+01
**GO_Biological_Process_2021**	negative regulation of transcription by RNA polymerase II (GO:0000122)	1.3E-03	5.0E-02	3.0E+00	2.2E+00	1.5E+01
**GO_Biological_Process_2021**	regulation of gene expression (GO:0010468)	1.3E-03	5.0E-02	3.0E+00	1.9E+00	1.3E+01

Supplemental Table 3: Summary table of donor information. Table summarising some covariates of interest by donor, such as sex, sequencing type, age bin (in years) and Fitzpatrick score. Some individuals were sampled more than once, however, no biological replicates were used in training. Any use of biological replicates in this document is stated in the body of the manuscript. Individuals are summarized by data cohorts, which are independent between them. All individuals were sampled using tape-stripping.

Characteristic	Data Cohort								
	All (n = 590)	ID1 (n = 210)	ID2 (n = 12)	ID6 (n = 83)	ID8 (n = 22)	ID11 (n = 8)	Banila et al (n = 174)	ID26 (n = 47)	ID27 (n = 34)
**Sex**									
**Female**	427 (72.4%)	112 (53.3%)	12 (100.0%)	75 (90.4%)	18 (81.8%)	3 (37.5%)	129 (74.1%)	47 (100.0%)	31 (91.2%)
**Male**	163 (27.6%)	98 (46.7%)	0 (0.0%)	8 (9.6%)	4 (18.2%)	5 (62.5%)	45 (25.9%)	0 (0.0%)	3 (8.8%)
**Age bin (Years)**									
**18-29**	120 (20.3%)	42 (20.0%)	0 (0.0%)	23 (27.7%)	2 (9.1%)	1 (12.5%)	52 (29.9%)	0 (0.0%)	0 (0.0%)
**30-39**	136 (23.1%)	50 (23.8%)	0 (0.0%)	18 (21.7%)	3 (13.6%)	5 (62.5%)	60 (34.5%)	0 (0.0%)	0 (0.0%)
**40-49**	76 (12.9%)	38 (18.1%)	0 (0.0%)	6 (7.2%)	5 (22.7%)	1 (12.5%)	21 (12.1%)	5 (10.6%)	0 (0.0%)
**50-59**	92 (15.6%)	30 (14.3%)	0 (0.0%)	19 (22.9%)	7 (31.8%)	1 (12.5%)	15 (8.6%)	5 (10.6%)	15 (44.1%)
**60-90**	166 (28.1%)	50 (23.8%)	12 (100.0%)	17 (20.5%)	5 (22.7%)	0 (0.0%)	26 (14.9%)	37 (78.7%)	19 (55.9%)
**Fitzpatrick**									
**I**	41 (6.9%)	18 (8.6%)	0 (0.0%)	3 (3.6%)	0 (0.0%)	0 (0.0%)	13 (7.5%)	5 (10.6%)	2 (5.9%)
**II**	244 (41.4%)	136 (64.8%)	6 (50.0%)	20 (24.1%)	10 (45.5%)	2 (25.0%)	53 (30.5%)	10 (21.3%)	7 (20.6%)
**III**	180 (30.5%)	48 (22.9%)	5 (41.7%)	25 (30.1%)	10 (45.5%)	3 (37.5%)	56 (32.2%)	15 (31.9%)	18 (52.9%)
**IV**	75 (12.7%)	8 (3.8%)	1 (8.3%)	11 (13.3%)	2 (9.1%)	3 (37.5%)	40 (23.0%)	7 (14.9%)	3 (8.8%)
**V**	39 (6.6%)	0 (0.0%)	0 (0.0%)	21 (25.3%)	0 (0.0%)	0 (0.0%)	7 (4.0%)	8 (17.0%)	3 (8.8%)
**VI**	11 (1.9%)	0 (0.0%)	0 (0.0%)	3 (3.6%)	0 (0.0%)	0 (0.0%)	5 (2.9%)	2 (4.3%)	1 (2.9%)

Supplemental Table 4: MitraClock's predictions for 12 Keratinocyte primary cell lines. Two of them, EM-Seq labial and eyelid keratinocytes, were processed in-house (Promocell, Heidelberg, Germany; lot 449Z024.1 and 491Z023.2) and the remaining 10 are derived from publicly available sources. The final 9 cell lines consist of NHEK (Keratinocyte) cell lines, with 3 controls and 6 NHEK-derived iPSC using two methodologies, D8 to KSR and TNT to KSR, with three replicates each (*Buckerry et al, Nature 2023*). The iPSC replicates show consistent age reduction compared to the control.

**Celltype**	**Accession**	**Sex**	**Age**	**MitraSolo**	**Δage**	**MitraCluster**	**Δage**
EM-Seq labial Keratinocyte	MitraBio	F	21	33.85	-12.85	32.54	-11.54
EM-Seq eyelid Keratinocyte	MitraBio	F	52	47.51	4.49	47.17	4.83
Epidermal-Keratinocytes-Z00000424	GSM5652321	M	26	32.2	-6.2	33.96	-7.96
NHEK iPSC (D8 to KSR)	GSM7445375	n/a	n/a	10.15	n/a	6.21	n/a
NHEK iPSC (D8 to KSR)	GSM7445376	n/a	n/a	10.07	n/a	6.40	n/a
NHEK iPSC (D8 to KSR)	GSM7445377	n/a	n/a	8.42	n/a	8.07	n/a
NHEK iPSC (TNT to KSR)	GSM7445378	n/a	n/a	9.14	n/a	6.44	n/a
NHEK iPSC (TNT to KSR)	GSM7445379	n/a	n/a	10.62	n/a	3.96	n/a
NHEK iPSC (TNT to KSR)	GSM7445380	n/a	n/a	9.71	n/a	8.24	n/a
NHEK (Keratinocyte)	GSM7445381	n/a	n/a	51.60	n/a	43.70	n/a
NHEK (Keratinocyte)	GSM7445382	n/a	n/a	55.93	n/a	44.67	n/a
NHEK (Keratinocyte)	GSM7445383	n/a	n/a	50.56	n/a	57.94	n/a
